# P08 The missing links in the chain

**DOI:** 10.1093/rap/rkab068.007

**Published:** 2021-10-19

**Authors:** Aicha Bouraoui, Louise Daniels

**Affiliations:** 1 University College London Hospital, London, United Kingdom; 2 Barking Havering and Redbridge Hospitals, London, United Kingdom

## Abstract

**Case report - Introduction:**

Sciatica symptoms are common symptoms in the general population. Most frequently, patients and health professional tend to conservatively manage symptoms assuming that disc prolapses are the main cause. Herein we describe a case of 69-year-old who had delayed diagnosis of spindle cell carcinoma secondary to radiotherapy for previous cancer. We will outline lessons learnt across the whole system, including carer’s perspective.

**Case report - Case description:**

We present a case of 69-year-old woman who was referred to rheumatology with a 3-year history of worsening back pain and sciatica symptoms. She has a longstanding history of achalasia and previous ovarian cancer for which she had curative TAH and BFO and subsequent radiotherapy in 1997.

The patient had worsening right leg paraesthesia and neuropathic symptoms suggestive of sciatica which started in 2013. She was self-managing her symptoms and practised yoga and home-based exercise. She was subsequently seen by her GP, NHS community physiotherapy team, private physiotherapist, osteopath, the GP practice physiotherapist and orthopaedics. An MRI spine was performed which showed degenerative changes with no acute nerve root compression and she was re-assured that there was no indication for surgery to the spine.  Despite analgesia, regular physiotherapy and TENS device, her symptoms worsened with night pain and sleep disturbance, hence her rheumatology referral in 2016.

Clinical examination revealed right-sided buttock and sacral tenderness with right upper leg wasting, right knee flexion and ankle dorsiflexion weakness and reduced sensation over the right lateral thigh and in the first web-space. Hence, an MRI pelvis and NCS/EMG were arranged.

EMG findings were highly suggestive of acute, severe, postganglionic, axonal lesion of the right lower lumbosacral plexus/right sciatic nerve affecting predominantly tibial division/S1 myotome. Subsequent MRI spine and pelvis revealed a right-sided sciatic nerve tumour and urgent biopsy confirmed grade 2 malignant peripheral nerve sheath tumour consistent with spindle cell carcinoma.

Patient was referred to the specialist sarcoma service MDT and had right leg and hemipelvis amputation. She unfortunately had an eventful post-op recovery with a number of complications, including pneumonia, stroke and small bowel obstruction secondary to metastatic disease and was referred to end of life care.

**Case report - Discussion:**

Back pain and sciatica are common symptoms, mostly secondary to disc prolapse or degenerative spine disease. The differential of causes is wide and it is vital to consider red flags. In this case previous history of cancer and radiotherapy should have alerted clinicians to do detailed examination and arrange relevant investigations accordingly.

Differentiating sciatic neuropathy from L5 or S1 radiculopathies or lumbosacral plexopathy can be difficult. With L5 or S1 radiculopathies, patients commonly experience low back pain radiating into the lateral or posterior leg (L5/S1 distributions). The pain in radiculopathy tends to improve with standing and walking and worsen with sitting. Whereas sciatic neuropathy typically start at the buttock area or proximal thigh and pain radiates posteriorly and laterally into the leg. Depending on severity, patients with a sciatic neuropathy can experience extreme weakness of knee flexion and movements of the feet. Sensation can be lost in lateral knee, lateral calf, dorsum of the foot, web space of the great toe, posterior calf and lateral foot and sole of the foot radiating posteriorly and laterally into the leg. Detailed clinical examination along with neurophysiological study would help to identify the level of the lesion.

Radiation-induced sarcoma (RIS) is a known long-term complication of radiotherapy, with an incidence ranging from 0.03% to 0.80% and latency of onset of 10 years or greater after treatment.

The definition of RIS has never been well established. Cahan criteria modified by Arlen et al. are most commonly used to define RIS: treatment with therapeutic irradiation at least 3 years prior to development of sarcoma, a sarcoma arising within the field of previous therapeutic irradiation; and differing histology between the sarcoma and the primary tumor.

Compared to other spontaneous cancers, spindle cell carcinoma is more aggressive and has poor prognosis with an overall of 5-year.

**Case report - Key learning points:**

- Clinicians need to assess for red flags in patients with back pain and sciatic symptoms including history of radiotherapy.
- It is important to differentiate between L5/S1 radiculopathy and sciatic neuropathy when assessing patients with sciatica symptoms.
- Patients receiving radiotherapy need to be made aware of long-term risks of secondary cancers and require regular follow up.

